# Basic moral sensitivity, moral disengagement, and defender self-efficacy as predictors of students’ self-reported bystander behaviors over a school year: a growth curve analysis

**DOI:** 10.3389/fpsyg.2024.1378755

**Published:** 2024-06-19

**Authors:** Björn Sjögren, Robert Thornberg, Jingu Kim, Jun Sung Hong, Mattias Kloo

**Affiliations:** ^1^Department of Behavioral Sciences and Learning, Linköping University, Linköping, Sweden; ^2^Behavioral Science Institute, Radboud University, Nijmegen, Gelderland, Netherlands; ^3^School of Social Work, Wayne State University, Detroit, MI, United States

**Keywords:** peer victimization, bullying, bystander behavior, basic moral sensitivity, moral disengagement, defender self-efficacy, longitudinal design

## Abstract

Though school children tend to view peer victimization as morally wrong most do not to intervene on the victim’s behalf and some instead choose to aid the victimizer. The aim of this longitudinal study was to investigate how students’ defending and pro-aggressive bystander behaviors evolved over the course of one school year and their association to basic moral sensitivity, moral disengagement, and defender self-efficacy. Three-hundred-fifty-three upper elementary school students (55% girls; 9.9–12.9 years of age) each completed self-report surveys at three points during one school year. Results from latent growth curve models showed that pro-aggressive bystander behavior remained stable over the year, whereas defending behavior decreased. Moreover, students who exhibited greater basic moral sensitivity were both less likely to be pro-aggressive and simultaneously more likely to defend. Students with defender self-efficacy were not only associated with more defending behavior at baseline but also were also less likely to decrease in defender behavior over time. Conversely, students reporting a higher degree of moral disengagement were linked to more pro-aggressive behavior, particularly when also reporting lower basic moral sensitivity. These short-term longitudinal results add important insight into the change in bystander behavior over time and how it relates to students’ sense of morality. The results also highlight the practical necessity for schools to nurture students’ sense of morality and prosocial behavior in their efforts to curb peer victimization.

## Introduction

Instances of peer victimization are typically observed by peers who are neither the perpetrators nor the victims (Atlas and Pepler, 1998; O’Connell et al., 1999), commonly referred to as bystanders (Salmivalli, 2014). While school children generally disapprove of acts of peer victimization (Eslea and Smith, 2000; Thornberg et al., 2017), bystanders do not always choose to intervene on behalf of the victims (defending bystanding) and, in some cases, may even intervene on behalf of the perpetrators (pro-aggressive bystanding), as noted by previous studies (Salmivalli et al., 1996; Gini, 2006; Pouwels et al., 2016). Research indicates that bullying is more prevalent in classroom settings where bystanders tend to act pro-aggressively by supporting the perpetrators, whereas it is less common in settings where bystanders tend to defend the victims (Salmivalli et al., 2011; Nocentini et al., 2013; Denny et al., 2015; Saarento et al., 2015). Additionally, observational studies suggest that when bystanders rally behind the victims, they often successfully halt the victimization incident (Hawkins et al., 2001). Moreover, having supportive bystanders on the victim’s side may serve as a protective factor, buffering negative emotional and psychosocial maladjustment (Ma and Chen, 2019).

Several empirical studies conducted over the last few decades have provided a comprehensive understanding of the correlates associated with students’ bystander behaviors (e.g., Salmivalli, 2014; Ettekal et al., 2015; Lambe and Craig, 2020). However, the predominance of cross-sectional studies (Ma et al., 2019; Lambe and Craig, 2020) severely constrains knowledge about whether and to what degree these correlates actually predict bystander behaviors. As a consequence, there is a considerable amount of uncertainty regarding which correlates might serve as effective components in schools’ prevention efforts aimed at enhancing students’ inclination to defend victims. Drawing upon social cognitive theory, the current study investigated how defending and pro-aggressive bystanding developed over the course of a school year, and how their levels and changes were associated with basic moral sensitivity, moral disengagement, and defender self-efficacy.

### Basic moral sensitivity

According to the social information processing (SIP) model (Crick and Dodge, 1994), how children and adolescents perceive and understand a social situation they encounter or are involved in influences their behaviors in the situation. Their social-cognitive processing in social situations includes six steps: (1) encoding of (internal and external) cues, (2) interpretation of cues, (3) clarification of goals, (4) response access or construction, (5) response decision, and (6) behavioral enactment. This processing is rapid and occurs in interaction with their database, which refers to their latent mental structures constituted by their past experiences and processing of social information stored in their long-term memory (e.g., social schemas, acquired rules, and social knowledge). According to Arsenio and Lemerise (2004), the latent mental structures also include sociomoral structures such as prototypical schemas for morality (e.g., fairness, welfare, and intentional harm).

Basic moral sensitivity, as introduced by Thornberg and Jungert (2013), pertains to “an individual’s readiness in morally simple situations to recognize moral transgressions and their harming consequences toward others, a sensitivity related to aroused moral emotions such as empathy, sympathy, or guilt” (p. 476). While many social situations typically involve potential conflicts between different moral norms, thus being characterized as morally complex situations, Thornberg and Jungert (2013) argued that bullying can be viewed as a prototype of a morally simple situation. In such situations, it is easy to recognize the moral wrongness and become emotionally aroused without conscious effort, but rather as a result of rapid, automatic information processing (also see Arsenio and Lemerise, 2004). Despite the general tendency for children to condemn bullying and sympathize with the victims (e.g., Rigby and Slee, 1991; Eslea and Smith, 2000; Thornberg et al., 2017), this does not hold for everyone (Perren et al., 2012). Therefore, it is reasonable to assume that there is an individual variation in basic moral sensitivity. Due to differences in past experiences and sociomoral development, students vary in the extent to which a prototype of “doing harm toward a victim” has been developed into an understanding of moral transgression linked to feeling empathy for the victim in their long-term memory and in what extent sociomoral schemas are likely to be retrieved when they encode and interpret cues in peer victimization situations (Arsenio and Lemerise, 2004).

To the best of our knowledge, only two prior studies have delved into the connection between basic moral sensitivity in bullying and bystander behaviors, both using self-report scales. Thornberg and Jungert (2013) discovered that basic moral sensitivity, mediated by moral disengagement, exhibited a negative correlation with pro-aggressive bystanding and a positive correlation with passive bystanding and defending. In other words, students high in basic moral sensitivity were less inclined to morally disengage, and lower moral disengagement was, in turn, related to less pro-aggressive bystanding and greater defending and passive bystanding. Similarly, in a more recent study, basic moral sensitivity showed a positive relationship with defending (Jiang et al., 2022). In addition, two other studies have further extended these findings by examining the related concept of students’ sensitivity to everyday moral situations. These studies found a positive association with self-reported defending behaviors and a negative association with self-reported pro-aggressive behaviors (Brugman et al., 2023; Xie et al., 2023).

### Moral disengagement

Beyond basic moral sensitivity, moral agency requires motivational and self-regulatory mechanisms to translate moral convictions into actual behavior (Bandura, 2002). During self-evaluation, individuals typically respond to their behavior by either approving their adherence to moral standards or applying self-sanctions (e.g., feelings of guilt) when those standards are violated. According to Bandura (2002), however, people can disengage from their moral standards by employing social and psychological strategies that deactivate self-regulatory mechanisms, thereby diminishing or eliminating self-sanctions against immoral conduct. Thus, while basic moral sensitivity is about being attuned to the moral implications of one’s actions, moral disengagement is about disconnecting oneself from those implications to justify immoral actions. More specifically, Bandura (2016) identified eight disengagement mechanisms categorized into four loci based on their focus. The behavioral locus relates to how individuals reframe harmful actions in a more positive light through moral justification, the use of euphemistic language, or advantageous comparisons. The agency locus encompasses the ways in which individuals diminish their own role, either by displacement of responsibility or diffusion of responsibility. The effect locus involves the act of disregarding or distorting the consequences of one’s behavior by downplaying, denying, or ignoring the negative effects of the behavior. Finally, the victim locus includes the mechanisms through which individuals can avoid self-sanctions by dehumanizing or blaming the victims. About the SIP model (Crick and Dodge, 1994; Arsenio and Lemerise, 2004), individual differences in how inclined students are to activate moral disengagement mechanisms when encountering peer victimization situations can be understood as differences in latent mental structures that affect how they interpret cues, clarify their goals and decide how to act.

Empirical evidence indicates that students with a greater tendency to morally disengage are more likely to participate in pro-aggressive bystander behavior (Sjögren et al., 2020; Thornberg et al., 2020; Wu et al., 2023) and less likely to intervene in defense of victims (Gini et al., 2022; Tolmatcheff et al., 2022; Eijigu and Teketel, 2023; Wu et al., 2023). Importantly, these associations hold true whether studies utilize self-reports or peer-reports, highlighting the robustness of these findings across different methods of data collection (for meta-analyses, see Killer et al., 2019; Luo and Bussey, 2023). While an increasing number of studies have supported a longitudinal association between moral disengagement and bullying perpetration (for a review, see Thornberg, 2023), much less is known about whether moral disengagement predicts bystander behaviors over time. Only one prior study has explored whether moral disengagement predicts pro-aggressive bystanding (Troop-Gordon et al., 2019). This study identified a positive bivariate correlation between students’ level of moral disengagement in fall and their subsequent self-reported pro-aggressive behavior in spring both for boys and girls. However, in the regression model that included other predictors (e.g., fall pro-bullying, empathy, and popularity), the longitudinal link was significant for boys only. Regarding defending, findings have been mixed. One study indicated that moral disengagement negatively predicted self-reported defending (Doramajian and Bukowski, 2015), while two other studies suggested the association between self-reported defending and moral disengagement to be non-significant (Barchia and Bussey, 2011; Gini et al., 2022).

### Defender self-efficacy

Moral agency encompasses not only the restraint of inhumane behavior but also the proactive engagement in humane actions. However, the translation of moral intentions into actual behavior is not solely guided by individuals’ moral standards and their inclination to disengage from those standards. It is also strongly influenced by their belief in their capacity to successfully take action. In social cognitive theory, a central mechanism of human agency is self-efficacy, which refers to individuals’ beliefs in their ability to act in ways necessary to achieve a specific goal (Bandura, 1997). The fifth step in the SIP model is the response decision and includes outcome expectations and self-efficacy evaluation, which influence children and adolescents’ decisions about what to do and not to do in the situation (Crick and Dodge, 1994). High self-efficacy beliefs not only make individuals more inclined to strive toward their goals but also increase their persistence in overcoming obstacles and enhance their ability to cope with stressful scenarios (Bandura, 1997).

In the domain of bystander behavior in peer victimization, researchers have examined defender self-efficacy, which refers to students’ confidence in their ability to effectively intervene and support victims when faced with instances of peer victimization (e.g., Thornberg and Jungert, 2013). This self-efficacy is rooted in the belief that one’s actions can make a meaningful difference for those suffering from peer victimization, reflecting a proactive moral stance. Essentially, defender self-efficacy can act as a bridge between moral agency and actual behavior, empowering individuals to become active defenders.

Consequently, empirical evidence indicates that students with higher levels of defender self-efficacy are more inclined to intervene and defend victimized peers (Sjögren et al., 2020; Thornberg et al., 2020; Eijigu and Teketel, 2023; Wu et al., 2023). These findings remain consistent regardless of whether the research relies on self-reports or peer-reports, underscoring the strength of these results across various data collection techniques for a meta-analysis (see Ma et al., 2019; for a review, see Lambe and Craig, 2020). In contrast, studies examining the associations between defender self-efficacy and pro-aggressive bystanding have yielded mixed results, showing both weakly negative associations (Thornberg et al., 2020) and null associations (Pöyhönen et al., 2012). Importantly, few studies have explored the longitudinal connection between defender self-efficacy and students’ bystander behaviors. Two studies found that defender self-efficacy predicted subsequent self-reported (Gini et al., 2022) and peer-reported (van der Ploeg et al., 2017) defending, while another study (Barchia and Bussey, 2011) did not provide clear support for a longitudinal association. Despite a moderate partial correlation between defender self-efficacy at time 1 and subsequent self-reported defending at time 2—controlling for various factors such as grade, gender, aggression, and victimization—this link did not remain significant in the subsequent regression analysis.

### The current study

While existing research highlights moral disengagement and defender self-efficacy as crucial correlates of bystander behaviors (Lambe and Craig, 2020; Luo and Bussey, 2023), the majority of previous studies have been cross-sectional. Consequently, less is known about how moral disengagement and self-efficacy predict bystander behavior over time. Moreover, the few longitudinal studies that do exist have primarily relied on just two measurement points, often spaced 6–12 months apart, limiting our understanding of the short-term dynamics and developmental trajectories of these factors. In addition to moral disengagement, basic moral sensitivity is identified as another moral psychological concept that might significantly correlate with bystander behaviors, as suggested by previous research (e.g., Thornberg and Jungert, 2013; Jiang et al., 2022). Furthermore, despite scholars suggesting that students’ bystander actions may vary both between and within peer victimization episodes depending on the social context (Huitsing and Veenstra, 2012; Gumpel et al., 2014), previous studies examining this have found a moderate stability of bystander behaviors over time (e.g., Salmivalli et al., 1998).

The aim of the current study was to investigate how fourth to sixth grade students’ bystander behaviors evolved over a school year and how their baseline levels and trajectories were associated with their initial levels of basic moral sensitivity, moral disengagement, and defender self-efficacy. The focus on fourth to sixth graders is particularly pertinent as research indicates that bullying behaviors are most prevalent among students within this age range, both in Sweden (Friends, 2022) and elsehwhere (Due et al., 2005). During this period, children also experience rapid and significant developmental changes in various domains (i.e., cognitive, emotional, social, and physical) and begin to establish their identities and striving for independence (Sawyer et al., 2012) and peers increasingly become a stronger social influence on individuals’ development and behavior (Brown and Larson, 2009).

Given previous research suggesting that moral disengagement plays a more significant role in pro-aggression, whereas defender self-efficacy plays a more important role in defending (Thornberg and Jungert, 2013; Sjögren et al., 2021a), we examined moral disengagement as a predictor of pro-aggression, and defender self-efficacy as a predictor of defending. Because only two studies have explored basic moral sensitivity in bullying and demonstrated its relevance to bystander behaviors, it was included as a predictor in both models. Despite the scarcity of previous longitudinal studies, we hypothesized that pro-aggression is positively associated with moral disengagement and negatively associated with moral sensitivity while defending is positively associated with both defender self-efficacy and moral sensitivity.

Given that social cognitive theory (Bandura, 1986) emphasizes that behaviors result from interdependent associations among multiple factors, we also explored potential interaction effects. Specifically, we examined the interaction between moral disengagement and moral sensitivity for pro-aggression, and the interaction between defender self-efficacy and moral sensitivity for defending, areas not previously explored in existing research. For example, it is conceivable that students who are confident in their ability to effectively intervene and support victims when faced with instances of peer victimization, and simultaneously display high basic moral sensitivity, would be particularly inclined to defend victimized peers. Additionally, we included gender and immigrant background as control variables to mitigate the potential effects of exogenous factors in the estimated models.

## Methods

### Participants

We gathered data using a web-based questionnaire from students in the fourth, fifth, and sixth grades at three different waves throughout the school year. During the initial data collection in November/December, 502 students participated. By the second wave in February/March, 31 students had dropped out, and by the third wave in May/June, an additional 118 students had dropped out. This resulted in a final sample of 353 students (55% girls, age range = 9.9–12.9 years old, *M* = 11.77, *SD* = 0.29). For attrition analyses, we examined whether students who continued their participation from the first to the second wave, from the first to the third wave, and from the second to the third wave differed from those who dropped out in terms of their levels of pro-aggressive bystanding and defending. Independent *t*-tests revealed no significant differences in the first-wave levels of pro-aggressive bystanding and defending between those who continued and those who dropped out. Similarly, there was no significant difference in second-wave levels of pro-aggressive bystanding between remaining participants and dropouts. However, a significant difference emerged in defending during the second wave, with those who dropped out displaying lower levels compared to those who also participated in the third wave (*t* = 2.16 *p* = 0.03, *M*_remainers_ = 5.02, *M*_dropouts_ = 4.74). Nevertheless, this significant difference was small, as indicated by Cohen’s *d* of 0.20 (Cohen, 1988). The sample included students from different socioeconomic backgrounds (from lower to upper-middle socioeconomic status) and socio-geographic locations (from rural areas to medium and large cities), with data collected in eight different municipalities. Thirteen percent of the participants had an immigrant background, that is, were not born in Sweden or were born in Sweden of foreign-born parents.

### Procedure

Before commencing the research, ethical approval was secured from the Regional Ethical Review Board in Linköping, Sweden. Information about the study was communicated to school principals and teachers, who granted permission to access the classrooms. Both written informed parental consent and student assent were obtained from all participants. The students involved in the study completed a web-based questionnaire on tablets while at school, at three time points over a school year. Before answering the questionnaire, participants received standardized instructions and were assured that their involvement was confidential and voluntary. They were explicitly informed of their right to withdraw from the study at any time without the necessity of providing a reason. On average, it took participants 20–30 min to complete the questionnaire.

### Measures

The questionnaire comprised 10 scales. For the current study, we utilized five of these (presented in the following order): one with background questions (1), one on basic moral sensitivity (5), one on defender self-efficacy (6), one on moral disengagement (7), and one on bystander behaviors (8).

#### Bystander behaviors in peer victimization

Students’ bystander behaviors were measured by a 15-item seven-point scale ranging from 1 = *strongly disagree* to 7 = *strongly agree*, which had previously been shown to display good psychometric properties among Swedish students (e.g., Sjögren et al., 2021a,b; Thornberg et al., 2024). The scale was developed based on the eight-item Student Bystander Behavior Scale (SBBS), which has shown a robust internal structure across three dimensions: supporting bullying, passive observation, and defending, confirmed through both exploratory and confirmatory factor analysis (Thornberg and Jungert, 2013). The modifications were made to incorporate various subtypes within these bystander roles, enhancing the scale’s comprehensiveness. In the scale used in the current study, students were asked, “Try to remember situations in which you have seen one or more students victimizing another student (for example: teasing, mocking, threatening, physically assaulting, or excluding). What do you usually do?” Then followed 15 items: five depicting pro-aggressive bystanding (e.g., “I laugh and cheer the peer victimizers on”), five depicting passive bystanding (e.g., “I just walk away”), and five depicting defending (e.g., “I help the victimized student”). For this study, we focused on the 10 items about pro-aggression and defending. Students’ responses on these two subscales were averaged and used as composite scales for each time point. The scales were internally consistent, as shown by Cronbach’s alphas of 0.79–0.86 for pro-aggressive bystanding and 0.84–0.87 for defending across the three waves. Three separate confirmatory factor analyses, one for each time point, were run and provided adequate support for the two-dimensionality of the scale: χ^2^(34) = 62, *p* = 0.003, CFI = 0.99, RMSEA = 0.04, SRMR = 0.04 for time point 1; χ^2^(34) = 87, *p* < 0.001, CFI = 0.97, RMSEA = 0.05, SRMR = 0.04 for time point 2; and χ^2^(34) = 69, *p* < 0.001, CFI = 0.97, RMSEA = 0.05, SRMR = 0.05 for time point 3. The confirmatory factor analyses were estimated with the maximum likelihood estimation using the Satorra-Bentler correction to account for the non-normal distribution of data. Because previous research indicates that bystander roles are dimensional, that is, students’ bystander behaviors are likely to vary within and between peer victimization episodes (e.g., Huitsing and Veenstra, 2012; Frey et al., 2014; Gumpel et al., 2014), we considered the bystander roles as fluid and did not categorize participants as belonging to one of the roles (see also Levy and Gumpel, 2018), but focused on pro-aggressive bystanding and defending as continuous variables.

#### Basic moral sensitivity

A three-item scale measured to what degree students recognized the harmful effects of bullying and sympathized with victims (1 = *strongly disagree* to 7 = *strongly agree*) that had previously been shown to display good psychometric properties among Chinese and Swedish students (Thornberg and Jungert, 2013; Jiang et al., 2022). The scale asked students to rate to what degree they agreed with each item (“A person who is subjected to bullying suffers terribly”; “Bullying harms the victim for a very long time”; “I really feel sorry for the kids getting bullied”; Cronbach’s α = 0.79). Students’ responses to these six items were averaged and used as composite scales for basic moral sensitivity at the first time point.

#### Moral disengagement

Students’ propensity to morally disengage was measured by the Moral Disengagement in Bullying Scale (MDBS) (1 = *strongly disagree* to 7 = *strongly agree*) that had previously been shown to display good psychometric properties among students in Australia (Runions et al., 2019), France (Tolmatcheff et al., 2022), and Sweden (Thornberg and Jungert, 2014). The scale asked students to rate to what degree they agreed with each item. Example items include: “People who get teased do not really get too sad about it”; “If my friends begin to bully a classmate, I cannot be blamed for being with them and teasing that person too”; “If you cannot be like everyone else, you have to blame yourself if you get bullied.” Together, the items covered all four loci and eight mechanisms of moral disengagement. Students’ responses to the 17 items were averaged and used as composite scales for moral disengagement at the first time point. Cronbach’s alpha of 0.90 indicated that the scale was internally consistent. Confirmatory factor analysis for the first time point, with the global construct of moral disengagement as a general factor and the four loci subtypes (i.e., the behavioral locus, the agency locus, the effect locus, and the victim locus) as first-order factors, displayed adequate fit: χ^2^(115) = 258, *p* < 0.001, CFI = 0.92, RMSEA = 0.07, SRMR = 0.05. The confirmatory factor analysis was estimated with the maximum likelihood estimation using the Satorra-Bentler correction to account for the non-normal distribution of data.

#### Defender self-efficacy

Students’ self-efficacy beliefs to defend victims were measured by a six-item seven-point scale (1 = *strongly disagree* to 7 = *strongly agree*) that had previously been shown to display good psychometric properties among Swedish students (e.g., Sjögren et al., 2020, 2024). The scale started with “I feel that I am very good at…” followed by six items; two related to verbal victimization (e.g., “…telling students off when they call another student mean things”), two items related to physical victimization (e.g., “…helping students who are subjected to shoves, punches, and kicks”), and two items relating to relational victimization (e.g., “…getting a group to stop excluding another student”). Students’ responses to these six items were averaged and used as composite scales for defender self-efficacy at the first time point. Cronbach’s alpha of 0.85 indicated that the scale was internally consistent. A confirmatory factor analysis for the first time point, with the global construct of defender self-efficacy as a general factor and the three efficacy subtypes (i.e., efficacy beliefs related to verbal, physical, and relational victimization) as first-order factors, displayed adequate fit: χ^2^(6) = 21, *p* = 0.002, CFI = 0.99, RMSEA = 0.07, SRMR = 0.02. The confirmatory factor analysis was estimated with the maximum likelihood estimation.

### Statistical analyses

All analyses were conducted in R Studio using R version 4.3.2 (R Core Team, 2023). We integrated the two subscales of pro-aggression and defending and carried out a combined growth curve model. To account for the non-normal distribution of data of pro-aggression, we employed maximum likelihood estimation with the Satorra-Bentler correction.

Within the framework of latent growth curve modeling, the repeated observed variables were utilized to estimate the unobserved underlying trajectory defined by two latent growth factors: the intercept and the slope (Byrne and Crombie, 2002). Initially, an unconditional growth model was employed to estimate the intercepts and slopes based on the three repeated measures of pro-aggression and defending, respectively. The factor loading for the three measures on the latent intercept factor of pro-aggression and defending, respectively, was fixed at 1.0 to represent the initial starting point of the trajectories. To define the linear metric of time, the factor loadings for the slope were set at 0, 1, and 2. Subsequently, conditional models were estimated by extending the previous models to include predictors. For pro-aggression, predictors included basic moral sensitivity, moral disengagement, the interaction between basic moral sensitivity and moral disengagement, gender, and immigrant background. For defending, predictors included basic moral sensitivity, defender self-efficacy, the interaction between basic moral sensitivity and defender self-efficacy, gender, and immigrant background. The covariances between the intercepts and the slopes were estimated in both models. Furthermore, since the students were nested within classrooms, we accounted for this in our analyses by applying the cluster argument in the growth() function in the lavaan package when running the models. The estimated standard errors and confidence intervals for the parameters in the model thereby became accurate given the hierarchical structure of the data. In addition to the chi-square test statistics, we used the comparative fit index (CFI), the root mean square error of approximation (RMSEA), and the standardized root mean square residual (SRMR) by using the recommended cut-off points suggested by Hu and Bentler (1999): CFI > 0.95, an RMSEA <0.06, and an SRMR <0.08.

## Results

### Descriptive statistics and correlations

Descriptive statistics and bivariate correlation coefficients are presented in Table 1. Moral disengagement at wave 1 was significantly correlated with pro-aggressive bystanding at all waves and with defending at wave 1. Defender self-efficacy at wave 1 was significantly positively correlated with defending at all waves. Correlations of basic moral sensitivity at wave 1 with pro-aggression and defending were not statistically significant. The strongest correlations were the between-wave correlations of pro-aggression and defending, respectively.

**Table 1:** Inter-correlations, means, and standard deviations for study variables.

Measure	1	2	3	4	5	6	7	8	9
1. MD_T1_	–								
2. DSE_T1_	−0.15^**^	–							
3. BMS_T1_	−0.12^**^	0.25^***^	–						
4. PRO_T1_	0.48^***^	−0.09	−0.06	–					
5. PRO_T2_	0.37^***^	−0.06	−0.03	0.75^***^	–				
6. PRO_T3_	0.33^***^	−0.05	−0.00	0.61^***^	0.76^***^	–			
7. DEF_T1_	−0.12^*^	0.60^***^	0.08	−0.13^*^	−0.17^**^	−0.14^**^	–		
8. DEF_T2_	−0.04	0.47^***^	0.05	−0.06	−0.13^*^	−0.10	0.77^***^	–	
9. DEF_T3_	−0.07	0.43^***^	0.02	−0.07	−0.15^**^	−0.13^*^	0.72^***^	0.89^***^	–
*MD*	1.67	5.12	5.77	1.30	1.34	1.35	5.11	5.04	4.87
*SD*	0.66	1.11	1.30	0.62	0.53	0.62	1.27	1.29	1.28

*N* = 353; MD, Moral disengagement; DSE, Defender self-efficacy; BMS, Basic moral sensitivity; PRO, Pro-aggressive bystanding; DEF, Defending; T1, Time point 1; T2, Time point 2; T3, Time point 3; ^*^p < 0.05, ^**^p < 0.01, ^***^p < 0.001.

### Growth curve analysis

The unconditional model fit the data well [χ^2^(11) = 14.95, *p* = 0.19, CFI = 0.98, RMSEA = 0.03, SRMR = 0.04]. In the unconditional model, the slope of pro-aggression was not significant whereas the slope of defending was negative and significant, indicating that students’ overall levels of pro-aggressive behavior remained stable but that their levels of defender behavior decreased over the school year. Both intercepts and slopes displayed significant variance, indicating that there was significant variance across students, both regarding their initial values and their slopes of pro-aggression and defending.

**Figure 1:**
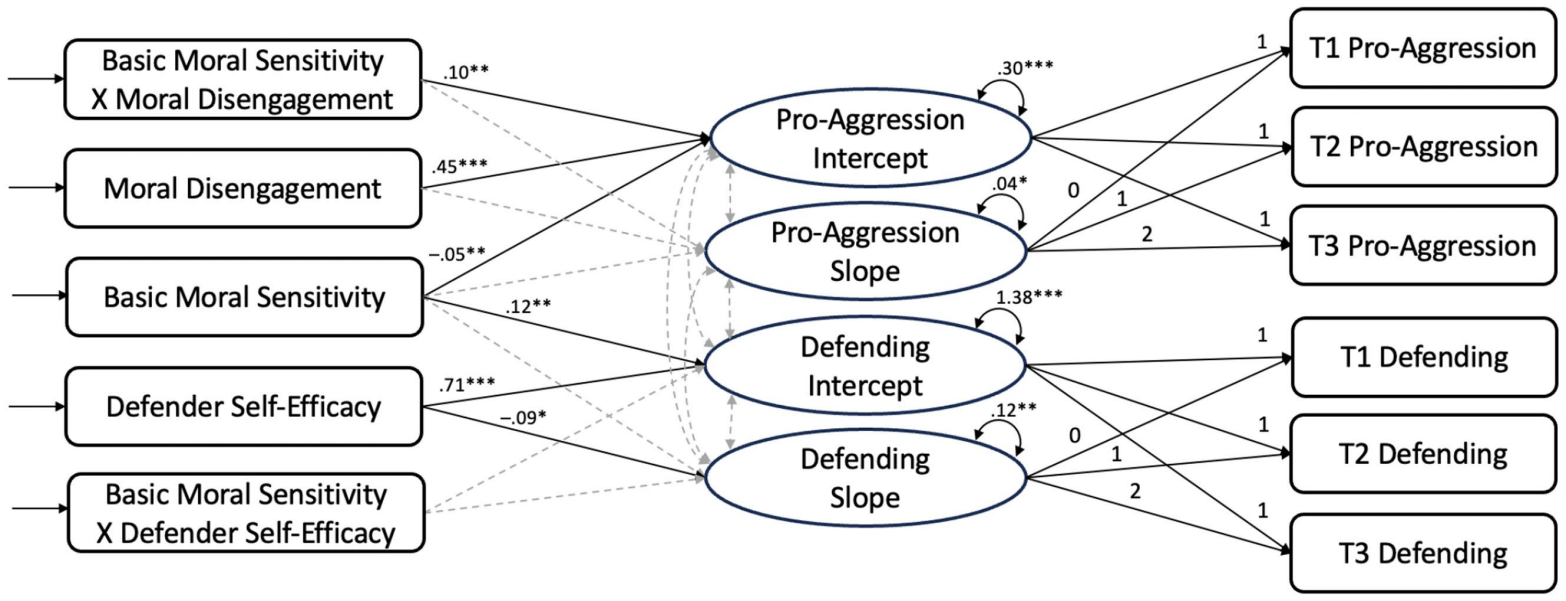
Latent growth curve model of pro-aggression and defending with unstandardized estimates. ^*^p < 0.05, ^**^p < 0.01, and ^***^p < 0.001. T1, Timepoint/wave 1; T2, Timepoint/wave 2; T3, Timepoint/wave 3. Nonsignificant estimates are indicated by dashed arrows. All effects of the control variables gender and immigrant background, not shown due to space limitations, were non-significant.

The conditional model that included predictors of pro-aggressive bystanding and defending fit the data well [χ^2^(33) = 45, *p* = 0.07, CFI = 0.98, RMSEA = 0.049, SRMR = 0.025]. For an overview and unstandardized estimates of the models, see Figure 1. Regarding the main effects of the conditional model, the results suggest that students had higher levels of baseline pro-aggression if they were high in moral disengagement (*Est* = 0.45, *p* < 0.001) and low in basic moral sensitivity (*Est* = −0.05, *p* = 0.007) at wave 1, whereas students had higher levels of baseline defending if they were high in defender self-efficacy (*Est* = 0.71, *p* < 0.001) and basic moral sensitivity (*Est* = 0.12, *p* = 0.002) at wave 1. Furthermore, students with higher levels of defender self-efficacy at wave 1 decreased less in defending over the school year (*Est* = −0.09, *p* = 0.020), compared to students with lower levels of defender self-efficacy at wave 1. Neither gender nor immigrant background was significantly associated with the intercepts or slopes of pro-aggression and defending.

**Figure 2:**
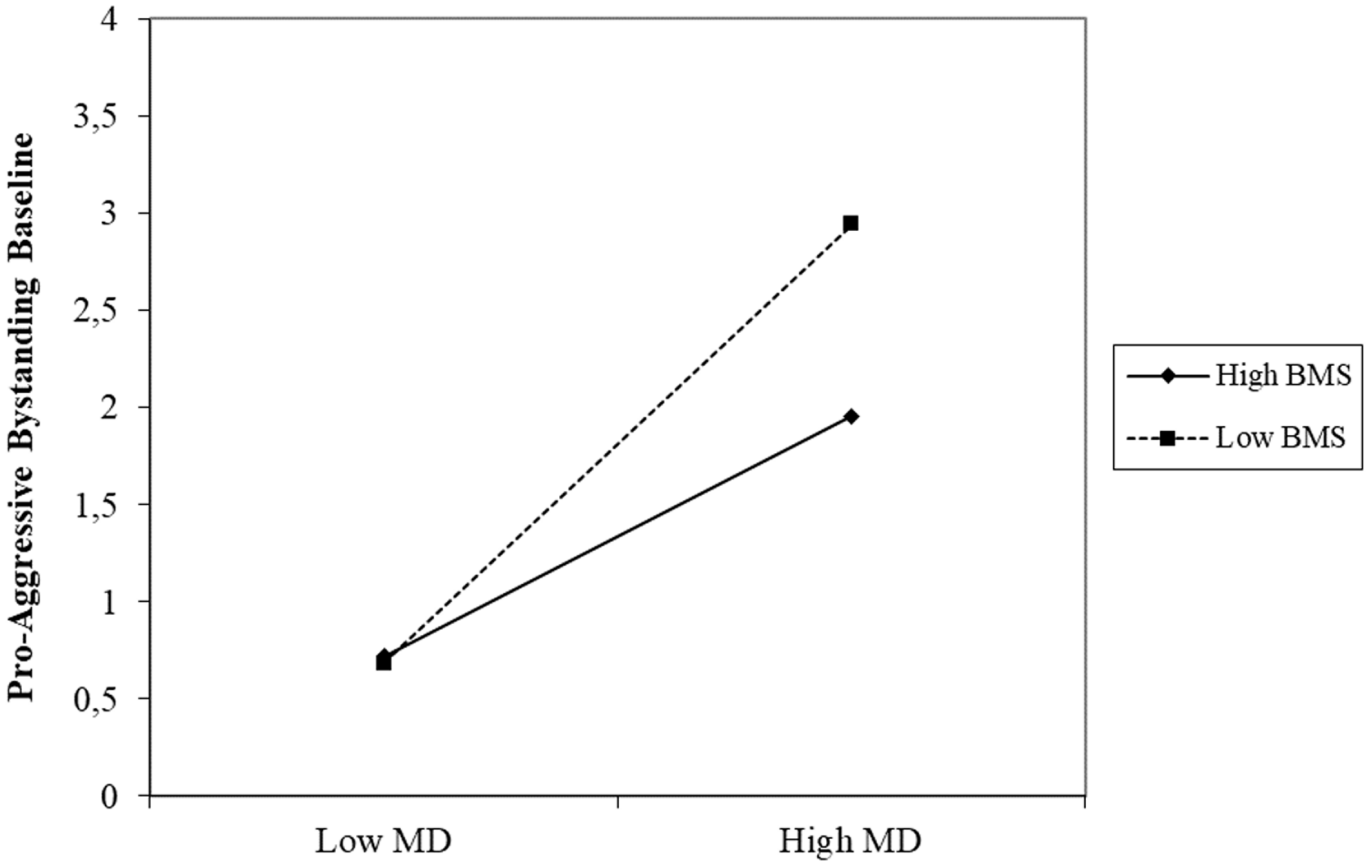
Interaction effect of basic moral sensitivity (BMS) and moral disengagement (MD) on pro-aggression.

Furthermore, we found a significant interaction effect between moral disengagement and basic moral sensitivity on the intercept of pro-aggression (*Est* = 0.10, *p* = 0.002). To interpret the significant interaction effect, we computed simple slopes (see Dawson, 2014) that estimated the association between pro-aggression and moral disengagement at low (one standard deviation below the mean) and high (one standard deviation above the mean) levels of basic moral sensitivity. The simple slopes for low and high levels of basic moral sensitivity were positive and significant (*Est*_low_ = 0.32, *p* = 0.004; *Est_high_* = 0.58, *p* < 0.001). Thus, although there was a general positive association between moral disengagement and pro-aggression, this association was more pronounced among students who were also low in basic moral sensitivity, as illustrated in Figure 2.

## Discussion

How students act as bystanders matters because peer victimization is less prevalent in classroom peer ecologies where students more often defend victims and less often side with victimizers when witnessing peer victimization (Salmivalli et al., 2011; Nocentini et al., 2013; Denny et al., 2015; Saarento et al., 2015). Even though research on students’ bystander behavior and its correlates has grown steadily during the past decades, most studies are still cross-sectionally, with only a small proportion of longitudinal studies (Lambe et al., 2019). The unique contribution of the present study was its longitudinal design including three waves over a school year and the investigation of how both students’ baseline levels and their trajectories of bystander behaviors were associated with basic moral sensitivity, moral disengagement, and defender self-efficacy, controlling for gender and immigrant background.

Drawing from the foundations of social cognitive theory (Bandura, 2016), moral agency consists of two fundamental forms: the capability to abstain from participating in inhumane conduct, which is termed inhibitive morality, and the aptitude to actively engage in compassionate and humane behavior, referred to as proactive morality. Previous empirical findings (e.g., Thornberg and Jungert, 2013; Killer et al., 2019; Lambe and Craig, 2020; Sjögren et al., 2021a; Luo and Bussey, 2023) have suggested that moral disengagement plays a significant role in explaining the inhibitive component (e.g., pro-aggressive bystanding), whereas defender self-efficacy plays a significant role in explaining the proactive component (e.g., defending). In addition, only a couple of studies (Thornberg and Jungert, 2013; Jiang et al., 2022) have investigated whether basic moral sensitivity is related to bystander behaviors. The findings are promising and point to a need to do further research to replicate and expand their findings.

### Pro-aggressive bystander behavior

Although students in general condemn bullying and sympathize with the victims (e.g., Rigby and Slee, 1991; Eslea and Smith, 2000; Thornberg et al., 2017), there is an individual variation in students’ levels of basic moral sensitivity. For example, even though Thornberg et al. (2017) found that bullies were inclined to judge bullying as wrong, they were less inclined to condemn bullying than their peers. Following previous studies examining the link between basic moral sensitivity and pro-aggressive bystander behavior (Thornberg and Jungert, 2013; Brugman et al., 2023; Xie et al., 2023), the current study found that students’ baseline levels of pro-aggressive bystanding were negatively related to their basic moral sensitivity. In other words, students who scored higher in basic moral sensitivity at the first wave were less likely to side with victimizers by assisting them or reinforcing their aggressive behaviors toward the victim across the three waves. This finding supports the theoretical understanding of basic moral sensitivity (Thornberg and Jungert, 2013; Jiang et al., 2022). Not only should high basic moral sensitivity make students more prone to judge bullying as wrong, see its harm, and recognize the victim’s distress and suffering, but also be more inclined to realize that pro-aggressive bystander behavior is a moral transgression in itself as it would add harm toward the victim.

Regarding Crick and Dodge’s (1994) SIP model, students encode and interpret the particular social situation and its social cues in the two first steps of the model. If they are high in basic moral sensitivity, we argue that they are more likely to notice moral and immoral cues and understand the moral transgression in a peer victimization situation and the harm it causes the victim. As a result of how they process social information, students high in basic moral sensitivity are more prone to feel empathic arousal and sympathy for the victim as bystanders (Hoffman, 2000; Arsenio and Lemerise, 2004) as well as perceiving transgressive guilt if they side with the perpetrators (Hoffman, 2000). Altogether, their moral basic sensitivity should produce a moral motivation to refrain from siding with the perpetrators and co-harming the victim by engaging in pro-aggressive bystander behaviors.

Students’ moral compass can, however, be set aside by moral disengagement mechanisms. Consistent with the social cognitive theory (Bandura, 2002, 2016) and previous empirical findings (Sjögren et al., 2020; Thornberg et al., 2020; Wu et al., 2023), the current study also showed that students’ baseline levels of pro-aggressive bystanding were positively related to moral disengagement. Thus, students who showed greater moral disengagement at the first wave were more likely to assist victimizers or reinforce their aggressive behaviors toward the victim across the three waves in their role as bystanders of peer victimization. According to the social cognitive theory (Bandura, 2016), individuals constantly engage in self-evaluation in which they align their actions to their behavior by either approving their adherence to moral standards or applying self-sanctions such as feelings of guilt and shame when those standards are violated. However, children who are more prone to activate moral disengagement mechanisms can avoid moral self-sanctions and would, therefore, be at a higher risk of engaging in pro-aggressive bystanding, since they would be less inclined to perceive the witnessed peer victimization and their own behavior as wrong.

When comparing these two predictors, the beta coefficients in the model clearly showed that moral disengagement played a much stronger role than basic moral sensitivity in explaining the variation of pro-aggressive bystander behavior, even though both matter. In line with this, we found that students low in moral disengagement were less inclined to side with the victimizers independently of the levels of basic moral sensitivity. However, students high in moral disengagement were even more prone to engage in pro-aggressive bystander behavior if they were low in basic moral sensitivity than if they were high in basic moral sensitivity. Thus, having a high basic moral sensitivity seemed to partly suppress the inclination to assist perpetrators or reinforce peer victimization for students who scored high in moral disengagement.

It should be noted that none of the variables displayed a significant association with the trajectory of pro-aggressive bystander behavior. This observation contrasts with what one might have anticipated based on social cognitive theory and the one prior study that has examined and identified a longitudinal link from moral disengagement to pro-aggressive bystander behavior (Troop-Gordon et al., 2019). However, it is essential to consider that the level of pro-aggressive bystanding remained stable across all three-time points and that the slope variance was non-significant in the unconditional model. Rather than providing evidence that basic moral sensitivity and moral disengagement cannot serve as predictors of pro-aggressive bystanding, the non-significant associations should primarily be viewed in the context of the absence of explanatory power due to the stability of pro-aggressive bystanding over the 6 months spanning from the first to the third wave of data collection.

### Defending behavior

The present findings did not only show that greater basic moral sensitivity was related to less pro-aggressive bystander behaviors among the students. It was also found to be positively associated with defending. In other words, students who were high in basic moral sensitivity in the first wave were more likely to defend the victim if they were witnessing bullying across the three waves. This result is in line with the few previous cross-sectional studies that have examined this link (Thornberg and Jungert, 2013; Jiang et al., 2022). A possible explanation for these findings is that students who score higher in basic moral sensitivity are more prone to perceive and interpret a bullying situation they witnessing as a serious and harmful bullying situation in which the victim suffers or is in distress. Not only could this be linked to the two first steps in the SIP model (Crick and Dodge, 1994) discussed above, but also to Latané and Darley’s (1970) decision model of bystander intervention, in which the bystander must notice that something is wrong and define the event as an emergency (i.e., interpret a need for help) as necessary steps toward helping the victim. Regarding the SIP model (Crick and Dodge, 1994; Arsenio and Lemerise, 2004), if students have developed a clear moral understanding that peer victimization is an unfair moral transgression that causes harm and suffering as latent mental structures and if these structures are easily accessible, they would be activated and guide students’ social information processing when they become bystanders of peer victimization.

Because of their stronger proneness to notice and recognize that the peer victimization they are witnessing causes the victim harm, suffering, and distress, students high in basic moral sensitivity should be more inclined to experience moral emotions such as empathy and sympathy for the victim, moral anger toward the bully, and guilt if they remain passive or side with the bullies (see Thornberg et al., 2015). This would altogether motivate them to intervene on behalf of the victim (Hoffman, 2000). Accordingly, previous research has shown that students who more often engage in defending when they are bystanders score higher in empathy (Zych et al., 2019; Deng et al., 2021), empathic anger (Lambe et al., 2017; Pozzoli et al., 2017; Trach and Hymel, 2020), and moral emotions more generally (Thornberg et al., 2015; Trach and Hymel, 2020). However, intervention in peer victimization situations is risky because bullies, in particular, are often aggressive, powerful, and high in perceived popularity (Pouwels et al., 2016, 2018). Bystanders might, therefore, be inhibited by fear of social blunders, retaliation, and being the next victim, and perceive that they are not capable of doing anything to help the victim and stand up against the victimizers, which in turn demotivate them to intervene, even though they perceive the situation as wrong and think that they should help the victim (Forsberg et al., 2018; Spadafora et al., 2020; Strindberg et al., 2020). This leads to the importance of defender self-efficacy.

As we hypothesized, the current study revealed that defender self-efficacy was associated with students’ baseline levels and their trajectories of defending. These findings are consistent with social cognitive theory’s notion that self-efficacy beliefs constitute a central mechanism of human agency and that individuals with strong self-efficacy beliefs are more likely to persevere in their efforts to uphold moral standards and engage in prosocial behavior (Bandura, 1997). The findings are also in line with the SIP model assuming that children and adolescents’ self-efficacy evaluation affects their response decision in the social situation. More specifically, the findings suggest that students with stronger beliefs in their capability to intervene effectively were not only more inclined to engage in defending behavior initially but that they also decreased less in defending over the school year compared to students who possess lower levels of defender self-efficacy. This aligns with the few available longitudinal findings suggesting that higher levels of defender self-efficacy can predict defending (van der Ploeg et al., 2017; Gini et al., 2022). Although both basic moral sensitivity and defender self-efficacy were associated with defending in the current study, the findings stress that student’s beliefs in their capability to intervene effectively are more important than their responsiveness to moral transgressions in explaining the variability of defending.

### Limitations and suggestions for future research

Despite its strength, this study also has some limitations that need addressing in future studies. First, the current study did not consider the role of peer status in the longitudinal growth of bystander behavior. Previous studies have indicated that bystander behavior is often associated with peer status (Pouwels et al., 2018). Students high in basic moral sensitivity and defender self-efficacy are probably in a better social position to defend the victim if they have higher social status. High popular students might, however, be more prone to act as pro-aggressive bystanders if they are low in basic moral sensitivity to maintain their high social status by siding with powerful and high status bullies. In contrast, youth with marginalized peer status are probably more likely to have a low defender self-efficacy. Thus, even if they are high in basic moral sensitivity, they might be more inclined to be passive bystanders to minimize the risk of victimization while they might be more prone to act as pro-aggressive bystanders if they are high in moral sensitivity. Future studies should include passive bystanding and investigate the role of peer status for all three types of bystander behavior.

Second, the current research focused on individual-level predictors of bystander behaviors. In addition to individual-level factors influencing bystander behavior, the classroom social context is likely to play a significant role in its growth. For example, previous studies have shown that peer victimization is more prevalent in classrooms that score higher on hierarchical inequality (Garandeau et al., 2014; Babarro et al., 2017; Pan et al., 2020) and collective moral disengagement (Kollerová et al., 2018; Bjärehed et al., 2021). In contrast, peer victimization has been found to be less prevalent in classrooms that are higher in equality (Garandeau et al., 2014; Babarro et al., 2017; Pan et al., 2020) and cohesion (Babarro et al., 2017), with a more supportive, caring, friendly and respectful climate among the peers (Košir et al., 2020; Dietrich and Cohen, 2021; Thornberg et al., 2022), and where teachers are warmer and more caring and supportive (Di Stasio et al., 2016; Dietrich and Cohen, 2021; Kloo et al., 2023). Therefore, future studies need to take the sociomoral atmosphere of the classroom into account when examining individual variables and bystander behaviors, because it might also influence and motivate students who witnessing peer victimization to be more inclined to take different bystander actions. Due to the classroom context, students might as bystanders have concerns about their own social status and vary in fear of being attacked or victimized and their belief in their ability to intervene, whether they perceive the aggression as wrong, and in what ways they attribute cause and responsibility, which has been suggested in qualitative studies examining students’ perspectives (Forsberg et al., 2014, 2018; Chen et al., 2016; Strindberg et al., 2020), Accordingly, previous work has indicated that bystander behaviors are contingent upon classroom contexts. Students who are witnessing peer victimization in school are more likely to defend a victim if they belong to a classroom with a more supportive, caring, friendly and respectful climate among the peers (Thornberg et al., 2017), with a greater collective efficacy to stop peer aggression (Sjögren et al., 2020; Thornberg et al., 2020), that have stronger antibullying norms (Peets et al., 2015; Lucas-Molina et al., 2018; Thornberg et al., 2022), and in which bullies are relatively unpopular (Pouwels et al., 2019). In addition to having direct associations with bystander behaviors, and in accordance with social cognitive theory (Bandura, 1986, 1997, 2016), classroom variables can interact with individual variables in their influence on bystander behaviors (e.g., Gini et al., 2020). For example, morally disengaged youth tend to be more passive bystanders in classrooms where moral disengagement is prevalent (Thornberg et al., 2017). This contextual difference would influence both the initial level of bystander behavior and its growth over the course of the school year. Future studies should explore the contextual effects of classroom sociomoral context on students’ socio-cognitive characteristics and their bystander behaviors, and whether there are cross-level interaction and moderation effects.

Third, the current study examined the growth of bystander behavior and its predictors within a sample of middle childhood and early adolescence over a single school year. The role of friends and peers in the peer dynamics of bullying tends to be more salient in adolescence than in childhood. As peer influence plays a key role in bystander behavior, future studies could be replicated and extended by exploring different or broader age groups over a longer period.

Fourth, the current research captured changes in bystander behavior using a variable-centered approach. While this approach allowed us to investigate the longitudinal interplay among bystander behavior and its correlates, it is limited in capturing individual characteristics of youth in participant roles. For instance, defending bystanders could also be characterized as lacking basic moral sensitivity and defender self-efficacy. A person-oriented approach, such as latent profile analysis, could be used to capture the multiple combinations of bystander behavior and changes in profiles over time.

Fifth, the measures used in the current study consistently constituted of self-report seven-point Likert scales, administrated in a specific order for all participants. This methodology may have introduced biases related to social desirability, perception, and recall biases. Future studies could, among other things, include peer nominations and also explore similarities and differences between self-reported and peer-nominated bystander behaviors.

Lastly, the attrition analysis showed that students who dropped out after the second wave displayed lower levels of defending compared to those who continued their participation, thus suggesting that the missing data were not missing completely at random. However, the effect size of Cohen’s *d* was small, suggesting that although the observed difference was statistically significant, its practical impact should be limited. Still, if the reasons for dropout to some degree were related to the measured constructs, the results of the current study could still have been biased and future research should ideally strive to retain a higher participation rate.

### Practical implications

These limitations aside, findings from the study have implications for school-based practice. As indicated in the study, high moral sensitivity is related to students’ judgment of bullying as wrong, ability to perceive its harm, and recognition of the victim’s distress and suffering. Thus, practitioners need to consider intervention programs that increase students’ moral sensitivity and foster students’ prosocial behaviors. A program that is guided by the social–emotional learning (SEL) framework, which includes increasing positive youth development and promoting social and emotional competencies is one possibility. SEL-based programs are specifically aimed at fostering students’ awareness of the emotions and social situations of others, such as their peers. Although studies, to our knowledge, have not explored the role of SEL in students’ defending behavior, research has shown that SEL programs are promising in inhibiting bullying (e.g., Espelage et al., 2015). SEL programs could potentially increase students’ moral sensitivity, which would potentially cultivate prosocial behaviors.

The study also found that students who were high in moral sensitivity were likely to defend the victim in bullying situations. Given that defending behavior is found to be related to less bullying, practitioners must find ways to increase students’ moral sensitivity, and one way to do so is to increase students’ empathic responses. The negative correlation between empathy and students’ bullying and the effectiveness of empathy training intervention programs on students’ bullying has been well-documented in the research literature (Özkan and Gökçearslan-Çiftçi, 2009; Sahin, 2012). In addition, anti-bullying programs that are aimed at increasing students’ empathy and efficacy beliefs to defend victims are highly suggested. One potential program is the KiVa Anti-Bullying Program, which is aimed at prevention, intervention, and annual monitoring (KiVa Program, n.d.). According to one empirical study, KiVa is found to have a positive effect on students’ affective empathy (Garandeau et al., 2016). Targeting students’ affective empathy should be important as it reportedly is strongly associated with students’ defender self-efficacy (Lambe and Craig, 2020) and their likelihood of defending victims of bullying (Fredrick et al., 2020).

## Data availability statement

The raw data supporting the conclusions of this article will be made available by the authors, without undue reservation.

## Ethics statement

The studies involving humans were approved by Regional Ethical Review Board in Linköping, Sweden. The studies were conducted in accordance with the local legislation and institutional requirements. Written informed consent for participation in this study was provided by the participants’ legal guardians/next of kin.

## Author contributions

BS: Conceptualization, Data curation, Formal analysis, Investigation, Methodology, Project administration, Software, Visualization, Writing – original draft, Writing – review & editing. RT: Conceptualization, Funding acquisition, Writing – original draft, Writing – review & editing. JK: Resources, Validation, Writing – review & editing. JH: Resources, Validation, Writing – review & editing. MK: Investigation, Software, Validation, Writing – review & editing.
